# Processing of Ordinal Information in Math-Anxious Individuals

**DOI:** 10.3389/fpsyg.2021.566614

**Published:** 2021-04-21

**Authors:** Àngels Colomé, Maria Isabel Núñez-Peña

**Affiliations:** ^1^Section of Cognitive Processes, Department of Cognition, Development and Educational Psychology, Faculty of Psychology, University of Barcelona, Barcelona, Spain; ^2^Institute of Neurosciences, University of Barcelona, Barcelona, Spain; ^3^Section of Quantitative Psychology, Department of Social Psychology and Quantitative Psychology, Faculty of Psychology, University of Barcelona, Barcelona, Spain; ^4^Institut de Recerca Sant Joan de Déu, Barcelona, Spain

**Keywords:** math anxiety, ordinality, reverse distance effect, attentional control, cognitive flexibility

## Abstract

This study aimed to investigate whether the ordinal judgments of high math-anxious (HMA) and low math-anxious (LMA) individuals differ. Two groups of 20 participants with extreme scores on the Shortened Mathematics Anxiety Rating Scale (sMARS) had to decide whether a triplet of numbers was presented in ascending order. Triplets could contain one-digit or two-digit numbers and be formed by consecutive numbers (*counting condition*), numbers with a constant distance of two or three (*balanced*) or numbers with variable distances between them (*neutral*). All these triplets were also presented unordered: sequence order in these trials could be broken at the second (D2) or third (D3) number. A reverse distance effect (worse performance for ordered balanced than for counting trials) of equal size was found in both anxiety groups. However, HMA participants made more judgment errors than their LMA peers when they judged one-digit counting ordered triplets. This effect was related to worse performance of HMA individuals on a symmetry span test and might be related to group differences on working memory. Importantly, HMAs were less accurate than LMA participants at rejecting unordered D2 sequences. This result is interpreted in terms of worse cognitive flexibility in HMA individuals.

## Introduction

People who suffer from math anxiety have a feeling of tension and apprehension when they deal with number-related tasks in academic contexts or in their daily life (Richardson and Suinn, 1972). This emotional response is not unusual: according to the 2012 Program for International Student Assessment (Organization for Economic Co-operation and Development [OECD], 2013), 60% of 15-year-old students from OECD countries who were tested reported feeling worried about the difficulty of math tasks, and around 30% claimed they felt tense or nervous when they were solving math problems. This high prevalence is particularly worrying because math anxiety is negatively associated with mathematical learning and performance (Ashcraft and Faust, 1994; Organization for Economic Co-operation and Development [OECD], 2013), and math is one of the foundations of our highly technological societies. Therefore, in recent years, considerable effort has been devoted to investigating the factors that might cause the appearance and maintenance of math anxiety as well as its link with worse mathematical performance. An initial hypothesis was that highly math-anxious individuals [hereafter high math-anxious (HMA)] might have fewer working memory resources available when they perform math tasks because math-related ruminations would act as a secondary task, consuming part of them (e.g., Ashcraft and Kirk, 2001). We will come back to this hypothesis later in this introduction. A second hypothesis stated that HMA individuals might have less precise magnitude representation (Maloney et al., 2010, 2011), which would prevent them from learning more advanced math skills that are based on magnitude representation. Nevertheless, recent evidence has shown that it is not always the case that HMA individuals have worse numerical representations than their low math-anxiety peers (Dietrich et al., 2015; Colomé, 2019; Núñez-Peña et al., 2019). Concurrently, a change has taken place in the field of numerical cognition. The focus has shifted from research that was mainly on the cardinal meaning of numbers to a growing interest in ordinality (Lyons et al., 2016). Ordinality is a second property of numbers that refers to the relative position of an item in a sequence (e.g., first, second,…). It has been found that ordering abilities are related to mathematical performance, and it has been suggested that they might mediate between magnitude representation and arithmetic abilities (Lyons and Beilock, 2011) or may independently account for a considerable part of individual variance in arithmetic skills (Goffin and Ansari, 2016). Interestingly, there seems to be a switch over time in the relative contributions of cardinality and ordinality to achievement in mathematics (Lyons et al., 2014; Sasanguie and Vos, 2018). The increase in relevance of ordinality seems to be linked to the formation of a network of associative connections between numbers that are related through experience with either arithmetic or counting routines.

Our aim in this study was to investigate whether ordinal abilities in people with high math anxiety differ from their low math-anxious (LMA) peers. Given that math anxiety is often linked to worse arithmetic performance (e.g., Ashcraft and Kirk, 2001; Ashcraft and Moore, 2009) and considering the increasing connection between ordinality and mathematics achievement in a lifetime, we wondered whether one of the aspects in which HMA individuals might differ from their LMA peers is their ability to perform numerical order judgments. To the best of our knowledge, only one previous study has assessed this point: Douglas and Lefevre (2017), who explored the influence of basic cognitive skills on the relation between math anxiety and arithmetic performance, included order judgments between the numerical skills they assessed. Participants performed a timed paper-and-pencil task in which they had to decide whether a series of three numbers was ordered (ascending or descending) or not. Ordered series could contain consecutive numbers (*counting* condition, e.g., 2 3 4) or non-consecutive numbers (*neutral* condition, e.g., 2 5 7). Performance was calculated as the number of correctly judged items per second. Douglas and Lefevre (2017) reported a significant negative correlation (*r* = −0.28, *p* < 0.01) between this measure and the Abbreviated Math Anxiety Scale score (AMAS; Hopko et al., 2003), although they also found that the relationship between basic numerical skills and math anxiety was mediated by more complex skills. Nevertheless, given that their ordinality measure did not discriminate between conditions or between ordered and unordered trials, it is not very informative about the kinds of processes that HMA participants might perform differently from their LMA peers.

The signature of ordinal processing is the reverse distance effect (RDE). When cardinality is assessed through number comparison, a canonical distance effect is found in symbolic and non-symbolic magnitudes: the farther the numerosities are from each other, the easier it is to decide which one is larger (Moyer and Landauer, 1967). This effect has been attributed to the fact that numerosities are not represented exactly and there is a certain degree of overlap between close numerosities. In contrast, it has been observed (Turconi et al., 2006; Franklin et al., 2009) that participants who have to judge whether a triplet of numbers is properly ordered respond faster and more accurately to ordered series of consecutive numbers (e.g., 2 3 4) than to series of non-adjacent numbers (e.g., 2 4 6). This RDE is only found with symbolic materials, while non-symbolic ones exhibit the canonical distance effect (Lyons and Beilock, 2013).

Some authors (e.g., Bourassa, 2014; Vos et al., 2017) claim that the RDE might indicate the recognition of frequently encountered numerical sequences, with more familiar triplets being accessed faster and more accurately. Co-occurrence in daily life would lead to strong associations between these numbers in long-term memory networks, which would facilitate recognition and access to their positional information. In support of this hypothesis, Bourassa (2014) found, for instance, that ascending ordered sequences were answered faster than descending ordered ones. LeFevre and Bisanz (1986) also found that the recognition of series improved with very familiar, non-adjacent triplets such as 5 10 15. Familiarity would explain the RDE, since in general people are more exposed to consecutive sequences (as in counting routines) than to other sequences. Hence, it should be easier to judge *counting* triplets (e.g., 2 3 4) than *balanced* sequences, i.e., non-consecutive numbers with a constant distance between them (2 4 6). Moreover, both types of items should be easier than *neutral* trials, that is, triplets with non-consecutive numbers with unequal distances between them (3 4 6). Familiarity might account for individual differences, because individuals who deal with numerical symbols more frequently might have stronger, more sophisticated number associations stored. If familiarity is relevant in ordinal judgments, HMA individuals might be at a disadvantage because they tend to avoid math-related situations (Ashcraft and Ridley, 2005). For instance, Hembree (1990) showed that math anxiety was negatively related with measures such as the number of math courses taken at high school or the intention to enroll in college math. Given this avoidance, HMA individuals should have less practice with numbers, and fewer overlearned number sequences. Therefore, we expected that deciding whether a triplet was ordered would be harder for them in general, but particularly when sequences become more sophisticated such as in the balanced condition described above. However, the familiarity hypothesis has not always been supported. For instance, Lyons and Ansari (2015) found that RDE was stronger in triplets of two-digit numbers than in triplets of one-digit numbers, although the former type of numbers is less frequently encountered. This led them to conclude that ordinality is not just a consequence of other symbolic processes, such as familiarity with counting sequences, but a fundamental aspect of numerical processing. However, they acknowledged that the processes underlying ordinality judgment are currently largely unknown, and suggested some possibilities like verbal rehearsal, visuo-spatial processing, or serial-order working memory.

Indeed, individual differences in working memory are known to be relevant to the kind of associations between numbers that individuals establish. Lyons and Beilock (2009) asked participants to decide whether a triplet of numbers between 1 and 9 had been presented in ascending order. Triplets could be *sequential*^1^, when the triplet was a segment of the counting list (2 3 4), *balanced*, when numbers were not consecutive but had a constant distance between them (2 4 6), and *skewed*, if numbers had unequal intervals between them (3 4 6). Lyons and Beilock found that participants with high and low levels of working memory (hereafter HWM and LWM, respectively) found it hard to reject sequential triplets when they were unordered because the numbers contained in them were part of the overlearned counting sequence. Working memory groups did not differ in the *skewed* condition either: latencies and error rates were similar in the ordered and unordered trials of this condition, which indicates that there is a limit to the kind of ordinal associations that are made. Finally, *balanced* triplets behaved more like *sequential* triplets in the HWM group, while they showed a similar pattern to *skewed* triplets in the LWM group. Lyons and Beilock (2009) concluded that people with a high level of working memory build and retrieve deeper ordinal relationships than their LWM peers.

As we mentioned previously, working memory has also been invoked as one of the aspects that might be at the base of HMA individuals’ difficulties with mathematics (Ashcraft and Kirk, 2001). According to this hypothesis, HMA individuals ruminate about their poor math performance and their lack of abilities. This rumination acts as a secondary task that depletes the cognitive resources required for the math task in course. Therefore, lower WM capacity would not necessarily be a permanent consequence of HMA, but it may temporarily affect their resources available in mathematical situations. If this was the case, one might expect that the kind of ordinal associations they stored and retrieved were more superficial than those of their LMA peers. Therefore, we would expect, following Lyons and Beilock (2009), that anxiety groups did not differ in the counting and neutral conditions, but HMAs performed worse in the balanced trials.

In recent years, math anxiety has also been related to a deficit in attentional control that would make HMA individuals less efficient in numerical tasks (Suárez-Pellicioni et al., 2013, 2014, 2015). Attentional control refers to the human capacity to respond flexibly and in a goal-oriented way. We wondered if worse performance of HMA individuals in ordinality judgements might be related to this decrease in their processing efficiency and hypothesized that differences between math anxiety groups due to this deficit might be particularly noticeable in unordered triplets. As mentioned before, RDE is only found in ordered trials. In unordered trials, a canonical distance effect is usually obtained. This dissociation has been interpreted in the sense that both association-based and comparison strategies would be used when ordinality is judged. When there is a strong association between the numbers in the ordered triplet, direct retrieval of associations is faster and easier than the comparison process and may overrule it. However, decisions would be based by default on magnitude-comparison mechanisms. This would explain the distance effect found in unordered trials (Bourassa, 2014; Vos et al., 2017).

Unordered trials can consist of triplets in which the two initial numbers are ordered and the third one breaks the sequence (e.g., 3 4 2; hereafter the D3 sequence), and triplets in which the second digit is not in order (D2 sequence, as in 4 3 5). If participants must judge whether a sequence is in ascending order and the comparison of number pairs takes place sequentially, from left to right, D2 sequences should be discarded faster than D3 ones. Bourassa (2014) tested this hypothesis and found that her participants were faster in D2 than in D3 trials, so she concluded that they could apply a rejection rule to terminate the decision process quickly. Note that the third number was not completely ignored, since counting D2 sequences (5 3 4) were answered more slowly than neutral ones (5 4 7). Bourassa suggested that this might indicate an extra-check by participants or be caused by limitations in their strategic control. HMA individuals might have particular difficulties in applying this self-termination strategy: previous studies where an arithmetic verification had to be performed (Faust et al., 1996; Suárez-Pellicioni et al., 2013) found that HMA and LMA participants did not differ when the correct solution or a close incorrect alternative (e.g., 6 + 7 = 14) was proposed. Surprisingly, HMAs only showed difficulties—both in terms of flawed scores and electrophysiological response—when the proposed solution was clearly implausible (a split of 14 from the correct solution, as in 7 + 8 = 29). Suárez-Pellicioni et al. (2013) concluded that large-split solutions were unexpected in this context and had acted as distractors, making it harder for HMA participants to inhibit their extended processing. Similarly, it might be that HMA individuals who carry out an ordinal judgment cannot avoid performing exhaustive processing of the triplet, even if the first two numbers already indicate that the sequence is unordered. Other studies (e.g., Lemaire, 2010) have shown that aging, which is also characterized by weakened attentional control, leads to lower cognitive flexibility for switching from one strategy to another or inhibiting a given strategy.

To sum up, there are several reasons why HMA individuals might show low performance when they deal with ordinality tasks: (a) they often avoid mathematical situations and are less familiar with numbers, (b) math anxiety has been related to fewer available working memory resources in mathematical situations, which can lead to the storage of only shallow numerical associations, and (c) math anxiety is related to worse attentional control, which might lead to the use of inefficient strategies in ordinal judgements. However, ordinality has been little studied in people with high math anxiety. We aimed to reduce this gap with our study. In the experiment, we asked our participants to judge whether a triplet of numbers was presented in ascending order. Triplets of one- and two-digit numbers were used. Although Lyons and Ansari (2015) reported stronger RDE in two-digit triplets, we predicted that if HMAs are worse at judging ordinality because of their lack of familiarity with numbers, group differences might be stronger in the largest numbers, given that they are less frequent (Dehaene and Mehler, 1992).

We also manipulated the distance between numbers in the triplets, which could be consecutive (*counting sequence*) or at a constant distance of 2 or 3 (*balanced*). A third type of ordered trials (*neutral*) had varying distances (1–3) between the first and second numbers, and the second and third one: we expected that nobody would have these sequences that do not follow a pattern stored in their memories and hence, that no group differences would be observed on them. In contrast, and according to the literature, we predicted a main RDE in the ordered trials, that is, shorter latencies and more accurate responses to the counting sequence than to the balanced one. If HMA individuals are less efficient when they judge ordinality, RDE should interact with math anxiety and HMA participants might show a stronger RDE. Alternatively, one could compare each group’s behavior in every condition. If HMAs have a weaker or less sophisticated network of number associations in their long-term memory than their LMA peers, we would predict that only LMAs showed evidence of having balanced sequences stored in their memories. This was the pattern found in Lyons and Beilock (2009) when they compared participants with low and high working memory. Although lower WM resources related to math anxiety are supposed to be transitory, linked to the presence of intrusive thoughts in a mathematical situation, it was important to ensure that the two math anxiety groups did not also differ in their permanent working memory capacity. Therefore, as Lyons and Beilock (2009), we measured working memory of our participants through a span test and checked that any significant group effect obtained in the experimental task could not be related to different memory capacities. In the case of Lyons and Beilock (2009), a listening and a computation span were used. In our case, we opted for a symmetry span because the listening span was not directly applicable to our sample, since they were not English native speakers, and we thought that HMAs performance in the computation span might be contaminated by their anxiety toward numerical stimuli. In any case, authors such as Kane et al. (2004) have shown that all span tests reflect a domain-general factor.

Finally, we made use of unordered trials to investigate whether HMA participants are less flexible in the use of strategies and have more difficulties in self-terminating procedures. To achieve this, we created unordered trials in which the second item in the triplet was already unordered (D2) and trials in which only the third item broke the ascending sequence (D3). We predicted that if participants with high math anxiety are worse at strategy control, group differences would be particularly noticeable in D2.

## Materials and Methods

### Participants

Forty graduate students took part in the experiment. They were part of a larger sample tested for math and trait anxiety during an introductory psychology course who are included in the database of a longer project. Participants were divided into two groups of 20 based on their math anxiety scores. The LMA group consisted of 10 men and 10 women with a mean age of 23 years (SD = 2.6; age range = 20–29) who scored below the first quartile (score range = 30–54; mean = 42.6; SD = 6.9) on the Shortened Mathematics Anxiety Rating Scale (sMARS) (Alexander and Martray, 1989). The HMA group comprised 13 women and 7 men with ages ranging from 20 to 33 (mean age = 23.6; SD = 3.3). They scored above the third quartile on the sMARS (score range = 76–122; mean = 87.6; SD = 12.3). Both groups differed in their sMARS scores, *t*(38) = 14.28, *p* < 0.001, but not in age, *t*(38) < 1, or gender distribution, χ^2^(1) = 0.92, *p* = 0.33. Given that math and trait anxiety have been found to correlate (Hembree, 1990), we also ensured that the two groups did not differ in trait anxiety [*t*(38) = 1.30, *p* = 0.20; mean = 23; SD = 9.1, and mean = 27.3; SD = 11.8 for LMA and HMA participants, respectively]. All subjects were paid for their participation.

### Materials

#### Screening Phase

##### Shortened mathematics anxiety rating scale

The Spanish version of sMARS was used (Alexander and Martray, 1989; Núñez-Peña et al., 2013). sMARS is the 25-item version of the Math Anxiety Rating Scale (MARS) (Richardson and Suinn, 1972). Participants are presented with 25 situations that may cause them math anxiety (e.g., preparing to study for a math exam) and they use a five-point Likert scale to describe the level of anxiety caused by each item. The scale ranges from 1 (no anxiety) to 5 (high anxiety), so the sMARS total score can vary from 25 to 125. The Spanish version used here has strong internal consistency (Cronbach’s alpha = 0.94) and high 7-week test–retest reliability (intra-class correlation coefficient = 0.72).

##### State-trait anxiety inventory (STAI)

This tool has two scales that measure state (STAI-S) and trait anxiety (STAI-T) (Spielberger et al., 1983). Only the scale that assesses participants’ trait anxiety, i.e., their general tendency to respond with anxiety, was used here. It consists of 20 items describing different emotions. Participants must answer how they feel in “general” using a four-point Likert scale ranging from 0 (almost never) to 3 (practically always), so the scores range from 0 to 60. We used the Spanish version of the scale (Spielberger et al., 2008), which has excellent internal consistency (Cronbach’s alpha = 0.95), and adequate 20-day test–retest reliability with college students (*r* = 0.86).

#### Experimental Session

##### Order judgment task

Twenty-one triplets of one-digit numbers and 21 of two-digit numbers (range 18–53)^2^ were created and were distributed equally between counting, balanced, and neutral conditions (see Appendix A for a full list of the materials employed). All two-digit triplets contained a decade crossing, since previous studies (Franklin et al., 2009) had only reported RDEs in these kinds of items. When ordered, *counting* stimuli consisted of three numbers that were consecutive in the counting list (e.g., 3 4 5). *Balanced* triplets had a constant distance of two (four triplets) or three (three triplets) between the three numbers (2 4 6). Finally, numbers in the *neutral* condition had a distance of one, two, or three between each other, but the distance between the adjacent numbers was never the same (4 5 7).

Participants saw each triplet twice in an ordered way, and twice disordered: on one occasion (D2 sequence), the second number was larger than the first one, so participants did not need to identify the third number to decide that it was an unordered sequence (4 3 5). In the other type of disordered sequence (D3), participants needed to process the third number to provide a correct response (4 5 3).

One-digit and two-digit triplets were presented in separated blocks, and the order of presentation was counterbalanced across subjects. In each case, four blocks were created in which the whole 21 stimuli were randomly presented once and there was a similar number of ordered and unordered trials (10 vs. 11 trials of each). Each block was repeated twice for a total of 336 trials (7 triplets × 3 conditions × 3 sequence orders [but ordered sequences were presented twice] × 2 number of digits × 2 block repetitions).

##### Symmetry span task

In this automated test, participants performed a memory and symmetry judgment (Kane et al., 2004). Each trial began with the presentation of an 8 × 8 grid with some squares filled in white and others in black. Participants had to decide whether folding the image vertically would lead to two symmetrical halves. After responding, a 4 × 4 white grid appeared and one of the square cells was then filled in red. Participants were asked to remember the position of the colored square. The sequence symmetry and location to remember was repeated between two and five occasions. Finally, a new 4 × 4 white grid was presented and participants were asked to recall the position of the red squares in the same order in which they had appeared. There were 12 trials in total. The partial storage score was used, i.e., the number of correctly remembered items in the correct position, regardless of whether the entire trial has been retrieved correctly. This measure shows higher test–retest correlations, internal consistency, and correlation with other complex span tasks than absolute scores (Redick et al., 2012).

### Procedure

Participants were tested individually. On arrival at the laboratory, they were asked to sign a consent form and to fill in a questionnaire on demographic data. Subsequently, they were given the instructions: three numbers would appear on a screen and their task would consist of deciding whether they were in ascending order or not by pressing one of the two mouse buttons. Response buttons were counterbalanced across subjects.

Each trial had the following structure. First, a white-on-black fixation point appeared on the middle of the screen for 500 ms. Then, the triplet was presented until the participant responded or for a maximum of 2,500 ms. Finally, a blank of 500 ms was left between trials.

At the beginning of the experiment, all participants completed a practice block in which they received feedback on accuracy and latencies. The initial practice consisted of 10 trials comprised of triplets of two-digit numbers, other from those used in the experiment and similarly distributed across the three experimental conditions. Half of them were properly ordered and half were not.

Before the experimental session was concluded, participants’ working memory was measured using the Symmetry Span task (Kane et al., 2004).

### Data Analysis

We had three predictions, based on previous literature, on how ordinality judgments in HMA and LMA individuals might differ. First, we hypothesized that HMA might show a larger RDE than their LMA peers. Second, we raised the possibility that group differences were particularly strong in two-digit triplets, given that they are less familiar for everybody and in particular for HMA individuals, who try to avoid mathematical contents. In order to test these two predictions, we focused on the ordered trials: these items are the only ones in which the positions of the numbers in the triplets are respected and hence, the initial conditions (*counting, balanced*, and *neutral*) make sense. We first calculated the RDE for each participant by subtracting their accuracy rates and RTs in the balanced condition from the counting condition ones. To do so, we used the median latencies of the correct responses and accuracy rates. Medians were used instead of means because they are estimators more resistant to change with outliers (Maxwell and Delaney, 2004). Then, we ran two ANOVAs, one for accuracy and the other for latencies, with RDE as the dependent variable, number of digits (one and two) as within-subject factor, and math anxiety (LMA and HMA) as the between-subjects factor.

Even if similar sized RDEs were to be found in the HMA and LMA participants, we could not discard the possibility that there existed group effects in some or all the conditions. As we hypothesized in Section “Introduction,” HMAs might have weaker or less sophisticated number associations in their memory than their peers, either because of their tendency to avoid mathematical situations or because their worse memory resources when dealing with mathematical content. Therefore, we ran further analyses in which we compared group performance in each of the conditions. Given that two-digit numbers are less frequently encountered than one-digit ones and this could influence the kind of connection between numbers established, we also took this variable into consideration. Hence, we performed further ANOVAs on the accuracy rates and on the latencies of each condition (counting, balanced, and neutral), with number of digits (one and two) as within-subject variables and math anxiety as the between-subjects factor.

Last, our third prediction regarded the comparison of HMA and LMA behavior in the D2 condition, that is, the unordered sequences in which the second number in the triplet broke the order. Therefore, we ran an ANOVA for correct response latencies and accuracy rates on all the initial conditions together, with digits (one and two) as within-subject factor and math anxiety as a between-subjects variable. Subsequently, as a control and in order to ensure that group differences were exclusively found in the unordered sequences where the self-termination strategy could be applied, we also ran the same analysis on the D3 sequences.

## Results

### Symmetry Span Task

Participants in the LMA group had higher partial storage scores than their HMA peers [means of 34.4 and 29.8, respectively, *t*(38) = 2.12, *p* = 0.04]. Given that working memory and math anxiety scores were not independent, we were not able to introduce symmetry span as a covariate in subsequent analyses (Field, 2009) and provided a difference between math anxiety groups was found, we studied the effect of working memory separately.

### Ordinal Judgment Task

#### Ordered Trials: Latencies

There was a main effect of number of digits, *F*(1,38) = 57.24, *p* < 0.001, η*^2^_p_* = 0.66. Subsequently, we used one-sample *t*-tests to look at one and two-digit sequences separately: an RDE was found in both of them [*t*(39) = 3.74, *p* = 0.001 and *t*(39) = 12.74, *p* < 0.001, for one and two-digit triplets, respectively], although it was more than three times as large in the two-digit triplets (−53 vs. −172 ms), replicating the findings by Lyons and Ansari (2015). There was no main effect of math anxiety, *F*(1,38) = 1.11, *p* = 0.29, η*^2^_p_* = 0.02, and math anxiety did not interact with number of digits, *F*(1,38) < 1. In a second step, we compared performance of both groups in each condition: HMA and LMA participants did not differ in any of them and math anxiety did not interact with number of digits either (all *p*s > 0.13).

Table 1 shows the mean response times (RTs) and the proportion of hits for each number of digits, condition (counting, balanced, and neutral), and math anxiety group, as well as the mean RDE.

**TABLE 1:** Ordered triplets: mean reaction times (RT; standard error in brackets) and accuracy (ACC) for each number of digits, condition, and math anxiety group as well as size of the reverse distance effect (RDE).

	**LMA**				**HMA**			
	**Counting**	**Balanced**	**Neutral**	**RDE**	**Counting**	**Balanced**	**Neutral**	**RDE**
**One-digit triplets**								
RT	814 (43)	860 (50)	921 (54)	−46	902 (50)	963 (52)	1029 (64)	−61
ACC	0.96 (0.007)	0.94 (0.010)	0.93 (0.015)	0.02	0.92 (0.012)	0.90 (0.017)	0.92 (0.012)	0.02
**Two-digit triplets**								
RT	986 (48)	1141 (51)	1086 (49)	−155	1065 (48)	1253 (50)	1181 (53)	−188
ACC	0.96 (0.007)	0.91 (0.013)	0.93 (0.008)	0.05	0.96 (0.008)	0.87 (0.016)	0.91 (0.015)	0.09

#### Ordered Trials: Accuracy

**FIGURE 1:**
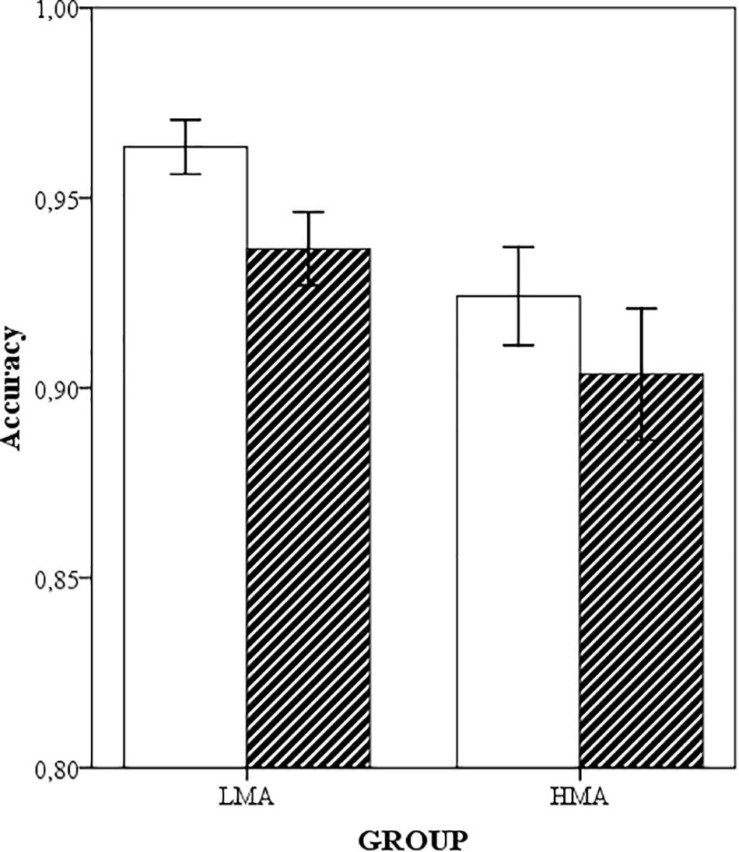
Means and standard errors (in bars) for the proportion of hits of each math-anxiety group in the one-digit counting (white) and balanced (striped) ordered conditions.

A main effect of number of digits was again observed, *F*(1,38) = 14.31, *p* = 0.001, η*^2^_p_* = 0.27. An RDE (see Figure 1) was found both in one-digit *t*(39) = 5.36, *p* = 0.026 and two-digit triplets *t*(39) = 46.01, *p* < 0.001, although its size was bigger for the second type of trials (2 vs. 7%). The size of the RDE did not depend on the math anxiety group, *F*(1,38) < 1, and math anxiety did not interact with number of digits, *F*(1,38) = 2.88, *p* = 0.098, η*^2^_p_* = 0.07.

Regarding performance in each particular condition, HMA and LMA participants performed similarly in the neutral one (*p* = 0.37). In contrast, LMA participants were more accurate than their HMA peers in the counting trials *F*(1,38) = 4.22, *p* = 0.047, η*^2^_p_* = 0.10. The main effect of number of digits and the digits × math anxiety interaction was also significant [both *Fs*(1,38) = 6.11, *p* = 0.018, η*^2^_p_* = 0.13]. Subsequent *t*-tests showed that HMA participants were significantly less accurate than their LMA peers in the counting one-digit trials only [*t*(38) = 2.65, *p* = 0.011; *t* < 1 for two digits]. As for the balanced trials, group effect was marginally significant, *F*(1,38) = 4.07, *p* = 0.051, η*^2^_p_* = 0.09 and math anxiety did not interact with number of digits (*p* = 0.75).

Therefore, and in contrast with what we predicted, the HMA group performed worse than the LMA one not in the balanced condition, where more sophisticated relationships were tested, but in the counting sequences. Our initial prediction was based on the findings of Lyons and Beilock (2009) on two groups differing in their working memory capacity. One of the most popular explanations of math anxiety (Ashcraft and Kirk, 2001) claims that it temporarily depletes part of the working memory resources necessary for performing the numerical task; therefore, we expected to replicate their results and find that our math anxiety groups differed mainly in the balanced condition. In contrast, we have found that group differences emerged even at the level of counting sequences. The fact that the difference between HMA and LMA accuracy in the balanced condition was only marginal could be due to the fact that even participants in our LMA group had developed weaker numerical connections than those on the LWM group of Lyons and Beilock (2009), because lower exposure to numerical situations or because they had lower working memory skills than their participants. Unfortunately, we did not use the same working memory test nor have any way to compare the exposure to numbers of both studies’ samples and these explanations remain speculative. In any case, given that our two math anxiety groups differed in their memory spans as measured by the symmetry test, we decided to verify whether the math anxiety effect was actually due to general differences in the working memory resources of the two groups. First, we correlated the mean accuracy rate in the one-digit counting condition with the symmetry span score and confirmed the existence of a significant positive correlation (*r* = 0.37, *p* = 0.02). Subsequently, a hierarchical stepwise regression was applied by taking accuracy in the counting sequences as the dependent variable and by entering into the model symmetry span and math anxiety scores (*r* = −0.29, *p* = 0.06) as predictors in step 1 and 2, respectively. The first model, including the WM score, accounted for 13% of the variance (*p* = 0.02). Adding math anxiety supposed an R change of 5%, which was not significant (*p* = 0.13). Subsequently, we also conducted a second hierarchical regression in which math anxiety score was entered in step 1 and WM span in step 2. On this occasion, the first model accounted for 8.7% of the variance (*p* = 0.06) and the second one, in which memory span was added as predictor, supposed a significant R change of 9.8%, *p* = 0.04. Although the second model including sMARS and WM span was significant (*p* = 0.02), WM span was the only significant predictor (β = 0.31, *p* = 0.04; β = −0.23, *p* = 0.14 for sMARS).

#### D2 Trials: Latencies and Accuracy

Concerning RTs, a main effect of number of digits was found *F*(1,38) = 90.85, *p* < 0.001, η*^2^_p_* = 0.70, with longer latencies for two-digit trials. The effect of math anxiety narrowly failed to reach significance, *F*(1,38) = 3.87, *p* = 0.056, η*^2^_p_* = 0.09, showing a trend for HMA being slower than their peers. As for accuracy, only the effect of math anxiety reached significance, with HMAs making more errors than their LMA peers *F*(1,38) = 4.72, *p* = 0.036, η*^2^_p_* = 0.11. Even if we had not *a priori* reasons to expect a relationship between performance in D2 trials and memory span^3^, we wanted to ensure that memory group differences were not playing a role in the D2 effects. As expected, the correlations between the symmetry span scores and the latencies and accuracy in D2 were not significant (*r* = −0.16, *p* = 0.30 and *r* = 0.28, *p* = 0.08, respectively).

**FIGURE 2:**
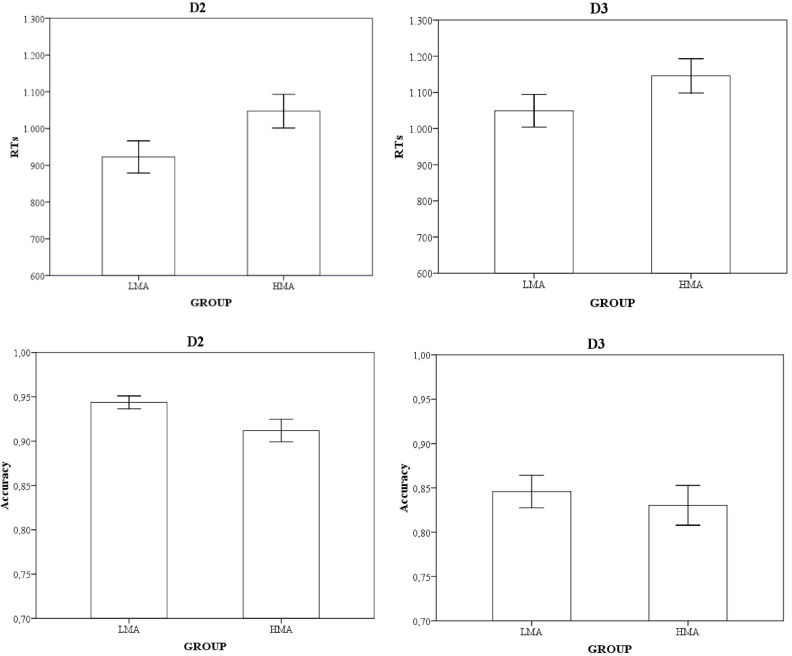
Means and standard errors (in bars) for reaction times (in ms and upper row) and the proportion of hits (lower row) for each unordered sequence (D2 and D3) and math anxiety group.

Figure 2 shows the mean latencies (RTs) and the proportion of hits for each unordered sequence (D2 and D3) and math anxiety group.

#### D3 Trials: Latencies and Accuracy

In order to ensure that group differences were exclusive to unordered trials that might benefit from self-termination, two new ANOVAs were conducted for D3 latencies and hit rates. In both cases, a number of digits effect was found, with better performance in the one-digit triplets [*F*(1,38) = 304.09, *p* < 0.001, η*^2^_p_* = 0.88, and *F*(1,38) = 16.79, *p* < 0.001, η*^2^_p_* = 0.30 for RTs and accuracy, respectively]. Math anxiety groups did not differ (all *p*s ≥ 0.15). Bayesian analysis performed with the JASP software (JASP Team, 2017) confirmed that the likelihood of a group effect was anecdotic: BF_10_ = 1.30 and BF_10_ = 0.47 for the latencies and accuracy analyses, respectively.

## Discussion

Ordinality has received increasing interest in recent years, especially since the finding that performance in ordinal judgments is a significant predictor of mathematical performance (Lyons and Beilock, 2011; Goffin and Ansari, 2016). However, to our knowledge, only one previous study (Douglas and Lefevre, 2017) had investigated ordinal performance in HMA individuals, albeit in the context of more general research on the influence of basic cognitive skills on arithmetic performance. Our study aimed to remedy this lack of knowledge by assessing ordinal judgment in two groups of participants with low and high math anxiety, in a variety of conditions. Participants had to judge whether triplets of numbers were presented in ascending order. The triplets were formed of one- or two-digit numbers, and the distance between the numbers in the triplet was manipulated. The break in order of the unordered triplets was in either the middle number (D2) or the last one (D3).

We replicated previous findings in the ordinality literature with a standard population. First, we observed an RDE with a decrease in performance, measured as reaction time and accuracy, as the distance between adjacent numbers in the triplet increased (Turconi et al., 2006; Franklin et al., 2009). Hence, it was easier for participants to judge triplets that constituted a segment of the *counting* sequence than triplets formed by numbers with a constant distance of 2 or 3 (*balanced condition*). Interestingly, we also replicated Lyons and Ansari (2015) finding of an interaction between number of digits and condition: although the RDE happened in both the one- and two-digit triplets, its size was three times as large in the two-digit items. As Lyons and Ansari (2015) claimed, this is hard to reconcile with ordinal judgments being largely driven by familiarity, since larger numbers are less frequent than smaller ones (Dehaene and Mehler, 1992).

We also investigated differences in order judgment associated with math anxiety. Math anxiety was related in previous studies to lower exposure to numerical stimuli (Hembree, 1990) and to fewer available working memory resources when dealing with number processing (Ashcraft and Kirk, 2001). We hypothesized that any or both of these factors might lead HMA individuals to have shallow associations between numbers. This might make it hard to recognize patterns such as fragments of the counting sequence, or other less familiar sequences such as those containing numbers with a regular distance between them. These difficulties might be particularly blatant in the less frequently encountered two-digit triplets. A third prediction, based on previous results by Suárez-Pellicioni et al. (2013), was that HMA participants would be less flexible and less prone to terminate the use of a given procedure when it was no longer necessary (D2 sequences).

Our data did not support the first two predictions. The RDE had an equivalent size in HMA and LMA participants in the one- and two-digit triplets. Nevertheless, we found some interesting group differences in the one-digit ordered triplets: HMA individuals were less accurate than their LMA peers even in the counting conditions. When we introduced the WM span as well as the sMARS score as possible predictors of our participants’ accuracy in the counting condition, we found that only the WM score was a predictor of their performance. Hence, it seems that the group differences found in this condition should be mainly attributed to variation in their general working memory resources. In any case, we cannot discard that other factors such as differences in familiarity with integers and the routinized rehearsal of certain sequences were also at the base of the math anxiety effects obtained here. It is a well-known fact (e.g., Hembree, 1990) that HMA individuals tend to avoid math-related situations and, although participants in the two groups were undergraduate psychology students with similar academic trajectories, their past and current contact with numeric content was not controlled. As the HMA group did not differ from their peers in the less familiar two-digit triplets, a relevant effect of familiarity alone is unlikely. However, considering our current data, this possibility cannot be rejected.

In contrast, our last prediction about the unordered series was fulfilled. When judging D2 triplets, individuals with HMA were significantly less accurate and tended to be slower than their LMA peers. A plausible explanation would be that they needed to terminate the exhaustive processing of the triplet even when it was no longer necessary. As mentioned previously, this behavior recalls that reported in Suárez-Pellicioni et al. (2013), in which HMAs had more difficulties than their LMA peers only when arithmetic verification of clearly implausible results was performed (i.e., additions with large-split solutions), which *a priori* should have been rejected very easily. This previous finding was interpreted within Attentional Control Theory (ACT, Eysenck et al., 2007), which claims that anxiety reduces attentional control by causing an imbalance in favor of the stimulus-driven attentional system and against the goal-directed one. This disparity has negative consequences on inhibition and shifting functions. Suárez-Pellicioni et al. (2013) took their results as an indicator that large-split solutions, which were unexpected, had acted as distractors and captured the attention of HMA participants, leading them to devote more resources to processing the solution. In the current study, D2 sequences cannot be considered as distractors, since they were formed by the same numbers as the other stimuli in the experiment. In contrast, what could explain the results in both studies within the framework of attentional control would be an incapacity for HMA individuals to flexibly implement the strategy required for a given stimuli and terminate its application when no longer necessary. Although it has been traditionally considered that anxiety would mainly reflect a tendency to distract and abandon the optimal procedures for reaching a goal, we consider that the opposite, that is, difficulty in disengaging from what initially was the proper strategy, might also be relevant. This cognitive rigidity has been observed in other populations. In Meiran et al. (2011), for instance, patients with obsessive compulsive disorders or unipolar depression showed slower disengagement from switching mode in subsequent single-task blocks than healthy controls. Interestingly, rumination scores in the Meiran et al. (2011) study correlated with poorer working memory updating, but they did not correlate with impaired fadeout from the switching mode, which replicates the dissociation between working memory functions obtained here, in which symmetry span scores were uncorrelated with performance on D2 sequences.

To sum up, the performance of HMA individuals in order judgment tasks was thoroughly explored for the first time. Although math anxiety effects were milder than predicted, two interesting differences with their LMA peers emerged. First, HMA participants were less accurate when they judged ordered counting one-digit triplets, although they performed similarly in the balanced, neutral, and all two-digit stimuli. This finding was related to differences in the updating memory function of the two anxiety groups. Second, HMA individuals found it harder to interrupt the processing of clearly unordered sequences. These data add to previous results that indicated that HMA people might have difficulties in terminating processing that is no longer relevant. It might be interesting to explore in further studies whether this lack of cognitive flexibility in HMA individuals is domain-general or specific to mathematical contexts.

## Data Availability Statement

The datasets presented in this study can be found in the OSF: https://osf.io/58m9q/?view_only=477f01cbb8514476bc284a6319154ab8.

## Ethics Statement

This study was reviewed and approved by the Ethics Committee of the University of Barcelona. The participants provided their written informed consent to participate in this study.

## Author Contributions

ÀC: conceptualization, methodology, software, investigation, formal analysis, and writing—original draft. MIN-P: conceptualization, resources, project administration, funding acquisition, and writing—review and editing. Both authors contributed to the article and approved the submitted version.

## Conflict of Interest

The authors declare that the research was conducted in the absence of any commercial or financial relationships that could be construed as a potential conflict of interest.

## Footnotes

1 *Sequential* and *skewed* conditions correspond, respectively, to the *counting* and *neutral* conditions in Douglas and Lefevre (2017) and the current work.

2 To ensure that the three conditions did not differ in other aspects such as the absolute size of the numbers involved (Franklin et al., 2009), we calculated the result of adding the three numbers in each triplet, and the mean of these additions for each condition did not differ, *F*(2,18) < 1.

3 Three functions of working memory have been postulated (Miyake et al., 2000): shifting, inhibition, and updating. Miyake et al. (2000) conducted a latent variable analysis on multiple tasks that are believed to tap working memory functions and concluded that operation span (and other analogous span tasks such as the symmetry one) only involved the ability to update and monitor incoming information. However, we considered performance on the D2 sequences as an indicator of participants’ attentional control, and therefore linked to the functions of inhibition and shifting, which are the two functions most affected by anxiety (Eysenck et al., 2007: see general discussion for further details).

## Appendix A | Triplets used in the experiment

**Table:**

	**One-digit triplets**			**Two-digit triplets**		
	**Counting**	**Balanced**	**Neutral**	**Counting**	**Balanced**	**Neutral**
Ordered	1 2 3	1 3 5	4 5 7	19 20 21	18 21 24	18 20 21
	2 3 4	3 5 7	2 3 6	18 19 20	19 21 23	18 19 22
	3 4 5	2 4 6	3 4 7	29 30 31	29 32 35	29 30 32
	4 5 6	4 6 8	4 6 7	28 29 30	28 30 32	28 29 32
	5 6 7	1 4 7	3 5 6	39 40 41	38 41 44	38 40 41
	6 7 8	2 5 8	2 5 6	38 39 40	39 41 43	39 42 43
	7 8 9	3 6 9	3 6 7	49 50 51	49 51 53	49 51 52
Unordered D2	2 1 3	3 1 5	5 4 7	20 19 21	21 18 24	20 18 21
	3 2 4	5 3 7	3 2 6	19 18 20	21 19 23	19 18 22
	4 3 5	4 2 6	4 3 7	30 29 31	32 29 35	30 29 32
	5 4 6	6 4 8	6 4 7	29 28 30	30 28 32	29 28 32
	6 5 7	4 1 7	5 3 6	40 39 41	41 38 44	40 38 41
	7 6 8	5 2 8	5 2 6	39 38 40	41 39 43	42 39 43
	8 7 9	6 3 9	6 3 7	50 49 51	51 49 53	51 49 52
Unordered D3	1 3 2	1 5 3	4 7 5	19 21 20	18 24 21	18 21 20
	2 4 3	3 7 5	2 6 3	18 20 19	19 23 21	18 22 19
	3 5 4	2 6 4	3 7 4	29 31 30	29 35 32	29 32 30
	4 6 5	4 8 6	4 7 6	28 30 29	28 32 30	28 32 29
	5 7 6	1 7 4	3 6 5	39 41 40	38 44 41	38 41 40
	6 8 7	2 8 5	2 6 5	38 40 39	39 43 41	39 43 42
	7 9 8	3 9 6	3 7 6	49 51 50	49 53 51	49 52 51
